# Genomic Stratification of Clozapine Prescription Patterns Using Schizophrenia Polygenic Scores

**DOI:** 10.1016/j.biopsych.2022.07.014

**Published:** 2023-01-15

**Authors:** Djenifer B. Kappel, Sophie E. Legge, Leon Hubbard, Isabella R. Willcocks, Kevin S. O’Connell, Robert L. Smith, Espen Molden, Ole A. Andreassen, Adrian King, John Jansen, Marinka Helthuis, Michael J. Owen, Michael C. O’Donovan, James T.R. Walters, Antonio F. Pardiñas

**Affiliations:** a MRC Centre for Neuropsychiatric Genetics and Genomics, Division of Psychological Medicine and Clinical Neurosciences, School of Medicine, Cardiff University, Cardiff, United Kingdom; b Magna Laboratories Ltd., Ross-on-Wye, United Kingdom; c Leyden Delta BV, Nijmegen, the Netherlands; d Norwegian Centre for Mental Disorders Research, Division of Mental Health and Addiction, University of Oslo and Oslo University Hospital, Oslo, Norway; e Section for Pharmacology and Pharmaceutical Biosciences, Department of Pharmacy, University of Oslo, Oslo, Norway; f Center for Psychopharmacology, Diakonhjemmet Hospital, Oslo, Norway

**Keywords:** Clozapine, Genomics, Pharmacogenomics, Polygenic risk scores, Precision medicine, Schizophrenia

## Abstract

**Background:**

Treatment-resistant schizophrenia affects approximately 30% of individuals with the disorder. Clozapine is the medication of choice in treatment-resistant schizophrenia, but optimizing administration and dose titration is complex. The identification of factors influencing clozapine prescription and response, including genetics, is of interest in a precision psychiatry framework.

**Methods:**

We used linear regression models accounting for demographic, pharmacological, and clinical covariates to determine whether a polygenic risk score (PRS) for schizophrenia would be associated with the highest dose recorded during clozapine treatment. Analyses were performed across 2 independent multiancestry samples of individuals from a UK patient monitoring system, CLOZUK2 (*n* = 3133) and CLOZUK3 (*n* = 909), and a European sample from a Norwegian therapeutic drug monitoring service (*n* = 417). In a secondary analysis merging both UK cohorts, logistic regression models were used to estimate the relationship between schizophrenia PRSs and clozapine doses classified as low, standard, or high.

**Results:**

After controlling for relevant covariates, the schizophrenia PRS was correlated with the highest clozapine dose on record for each individual across all samples: CLOZUK2 (β = 12.22, SE = 3.78, *p* = .001), CLOZUK3 (β = 12.73, SE = 5.99, *p* = .034), and the Norwegian cohort (β = 46.45, SE = 18.83, *p* = .014). In a secondary analysis, the schizophrenia PRS was associated with taking clozapine doses >600 mg/day (odds ratio = 1.279, *p* = .006).

**Conclusions:**

The schizophrenia PRS was associated with the highest clozapine dose prescribed for an individual in records from 3 independent samples, suggesting that the genetic liability for schizophrenia might index factors associated with therapeutic decisions in cohorts of patients with treatment-resistant schizophrenia.

Approximately one third of individuals with schizophrenia experience symptoms that do not meaningfully improve after 2 courses of standard antipsychotics, a presentation often called treatment-resistant schizophrenia (TRS) (1). Clozapine is the evidence-based treatment of choice for TRS (2), although it also has the potential to cause a range of adverse drug reactions (ADRs). These require careful clinical consideration and are major drivers of treatment discontinuation (3), contributing to the fact that most eligible patients are not offered clozapine as a treatment option (4). Moreover, it is estimated that only about 50% of those treated respond to clozapine (5), and few objective predictors of therapeutic response or adverse effects have been identified to date (6).

Individual differences in response to psychopharmacology are known to be influenced by genetic and environmental factors (7,8). Pharmacogenomics research aims to identify genetic variants that contribute to this variability and is one of the most promising pillars of precision medicine strategies (9). To date, while most known pharmacogenomic variants are associated with absorption, distribution, metabolism, and excretion processes influencing drug exposure, markers associated with disease and disorder risk can also be assessed to investigate treatment outcomes (10). In this sense, composite metrics of genetic risk such as polygenic risk scores (PRSs) have become widely used in medical genomics research and are also seen as potential predictive markers, which could eventually be introduced into patient care (11,12). As an example, hundreds of schizophrenia susceptibility loci have been identified in large-scale genome-wide association studies (GWASs) pointing to neurobiological pathways and mechanisms likely to be disrupted in the disorder (13). Several of these could feasibly play a role in antipsychotic treatment response, such as the dopaminergic signaling pathways indexed by *DRD2* (14). Thus, investigating the association between genetic liability for the disorder and response to antipsychotics might be fruitful, with the hypothesis being that a heavier genetic burden could be associated with poorer treatment response.

A key challenge for clinicians is determining the optimal dose of clozapine for a given individual, which requires weighing the relative likelihoods of therapeutic response versus ADRs. Clinical caution to avoid ADRs, which can be debilitating even if mild, might lead to individuals’ spending weeks or months on a given dose without apparent benefits before they are escalated to a higher one (15). In addition, meta-analytic evidence points to the need to take drug metabolism into account in clinical practice (16). In this sense, therapeutic drug monitoring (TDM) schemes, when available, can facilitate fine-tuning of clozapine concentrations (levels) for optimal response and are particularly suited for the identification of poor or rapid metabolizers (17), a subset of the general population that does not fully account for the rate of clozapine nonresponders (18). For these reasons, investigating clinical and demographic characteristics, including genetics, that underlie clozapine prescriptions in real-world settings can help us better understand the clinical decision-making processes behind clozapine dose escalation and provides a pathway toward the inference of predictive factors for treatment outcomes.

This study analyzed genetic and clozapine pharmacokinetic data in 3 retrospective cohorts: 2 from the CLOZUK project in the United Kingdom, one of the largest DNA sample collections worldwide of individuals with TRS (19), and another from the TDM service at Diakonhjemmet Hospital in Oslo (20). The aim was to assess whether the schizophrenia PRS would be correlated with the clozapine doses prescribed to those with TRS. We hypothesized that, if associated, schizophrenia PRS could indicate which patients would require higher doses of clozapine, and this information could be a proxy phenotype or indicator for poorer treatment response in the absence of ADRs. Given the underuse of clozapine and the complexities of its clinical management owing to dose-dependent and idiosyncratic ADRs, inferring the potential relevance of genomic information in this setting could be informative for the development of future stratification and drug dosing algorithms. In addition, novel observations supporting that schizophrenia genetic liability might also index therapeutic decisions and outcomes would be of great interest for precision psychiatry research.

## Methods and Materials

### Samples

The CLOZUK cohort consists of individuals taking clozapine in the United Kingdom whose DNA samples were collected anonymously. For this research, we accessed data from a subset of individuals termed CLOZUK2, which were linked to repeated assessments of clozapine pharmacokinetics. Additional descriptions of this cohort, genotyping procedures, and sample or data collection have been reported previously (19,21). A total of 3439 unrelated individuals over the age of 18 years were available from CLOZUK2 with genotypic data and more than 12,000 pharmacokinetic assays. This sample was curated to remove individuals taking clozapine for <18 weeks to ensure that steady-state levels of clozapine in plasma had been reached and to exclude individuals undergoing the initial titration process (15,22). In contrast to previous studies that have focused on European participants to minimize population stratification, we did not filter our data based on self-reported or genetically inferred ancestry. Our final curated dataset included a total of 3133 individuals from CLOZUK2. A summary of demographic and clinical characteristics is given in Table S1.

New in this study, we also report another wave of CLOZUK data, CLOZUK3, with more than 900 individuals and 5000 pharmacokinetic assays. Its collection follows the procedure detailed earlier for CLOZUK2 (19), including the curation protocol for clozapine-level data (21). For our analyses of CLOZUK3, we did not exclude individuals with clozapine treatment shorter than 18 weeks because treatment start date information was not available. Nevertheless, to increase compatibility with the curation procedures of CLOZUK2 and reduce the likelihood of analyzing individuals going through clozapine initiation/titration, we removed those in which the highest clozapine dose was <100 mg/day. Our final CLOZUK3 dataset included genetic and pharmacokinetic data for 909 individuals (Table S1).

Finally, we accessed data from a Norwegian cohort from the TDM database at the Center for Psychopharmacology at Diakonhjemmet Hospital Oslo, with 417 individuals linked to 7963 clozapine pharmacokinetic assays. This cohort included only Norwegian citizens of European ancestry and is fully described elsewhere (20). Phenotype data were extracted from TDM requisition forms filled out by clinicians, including information not explicitly available in CLOZUK, such as smoking habits and comedication profiles. From this information, we ensured that no Norwegian TDM samples showing concurrent use of clozapine with interacting drugs (e.g., fluvoxamine, a potent inhibitor of clozapine metabolism, or the potent enzyme inducers phenobarbital, phenytoin, and carbamazepine) were included in these analyses.

All procedures contributing to this work comply with the ethical standards of the relevant national and institutional guidelines (UK National Research Ethics Service approval [ref. 10/WSE02/15], following UK Human Tissue Act and Norwegian Regional Committee for Medical and Health Research Ethics approval [ref. 2014/1185]).

### Study Design

**Figure 1:**
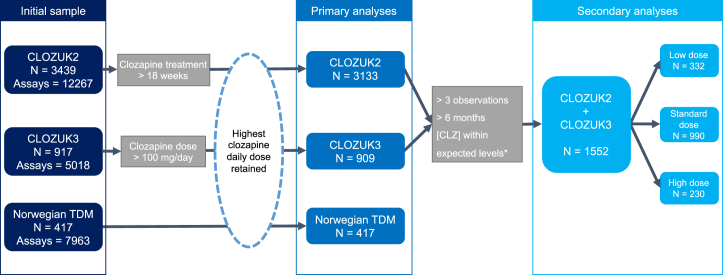
Sample inclusion flowchart. Curation procedures relevant to each analysis step for CLOZUK2, CLOZUK3, and the Norwegian samples are represented. ∗ indicates that dose-adjusted expected plasma levels were extracted from Couchman *et al.* (24). CLZ, clozapine; TDM, therapeutic drug monitoring.

For the primary analyses, we focused on the highest daily clozapine dose for each of the individuals included in our cohorts. Because sample collections, data curation, and genotyping procedures were carried out at different points in time for each dataset, we performed the analysis separately in CLOZUK2, CLOZUK3, and the Norwegian TDM cohort (Figure 1).

In secondary analyses, we merged the 2 CLOZUK cohorts because of their larger sample size and compatible phenotypic data and then stratified the individuals in these cohorts by their highest daily clozapine dose into 3 categories: 1) those taking a low dose (<300 mg/day), 2) those taking a standard maintenance dose (300–600 mg/day), and 3) those taking a higher dose than the usual maintenance dose (>600 mg/day) (23). For these analyses, we also included extra curation procedures (Figure 1). First, we selected only those individuals who had at least 3 assays in the clozapine monitoring system spanning a period of ≥6 months. This step was aimed at the clozapine dose we assessed to more accurately reflect the real highest dose that a participant was likely to have taken throughout their treatment. Second, we removed individuals likely not taking their medication (nonadherence) and/or presenting with an atypical (rapid/poor) clozapine metabolism because prescription patterns in these individuals would not likely follow the linear trends of the general population. This last procedure was done by excluding all assays in which the observed clozapine plasma concentrations did not match those expected for the recorded clozapine daily dose as reflected in Table 5 of Couchman *et al.* (24).

### Genetics

The genotyping of CLOZUK2 was conducted using Illumina HumanOmniExpress (Illumina, Inc.) arrays. A detailed description of genotyping, quality control, and imputation procedures for genomic data can be found elsewhere (19). The CLOZUK3 cohort was genotyped using the Illumina Infinium Global Screening Array-24 (Illumina, Inc.) and curated using the DRAGON-Data pipeline (25). For PRS analyses, imputed CLOZUK2 and CLOZUK3 dosages were converted to best-guess genotype calls (genotype probability > 90%, imputation quality metric [INFO] > 0.9, minor allele frequency > 10%, Hardy-Weinberg equilibrium mid *p* value > 10^−4^). The genotyping and imputation of the Norwegian cohort are also described in detail elsewhere (20).

For deriving the main predictor of interest, we computed genome-wide PRS profiles from the latest schizophrenia multiancestry meta-analysis from the Psychiatric Genomics Consortium (PGC) (13). Given that the CLOZUK2 and Norwegian cohorts were included in the analyses of this publication, to avoid sample overlap between training and testing sets, we derived deduplicated schizophrenia summary statistics before calculating the PRS in each sample. We only used summary statistics from the full PGC GWAS as a training set to derive PRS in CLOZUK3 because that sample was not included in the PGC meta-analysis. Because data within the CLOZUK cohorts do not include known predictors of demographic and lifestyle factors associated with drug metabolism (26), we also computed proxy PRSs for coffee intake, body mass index, and smoking behavior using summary statistics from the Genetic Investigation of Anthropometric Traits (GIANT) consortium (27) and the GeneATLAS UK Biobank GWAS resource (28). PRSs were computed using the PRS continuous shrinkage method (29), adjusted for linkage disequilibrium structure with default options and a shrinkage parameter of ϕ = 1 for schizophrenia (30) and ϕ = auto otherwise. Before statistical analysis, PRSs were standardized within each sample (mean = 0, SD = 1) to facilitate the interpretability of the results.

### Statistics

#### Primary Analyses

To analyze the association between the schizophrenia PRS and the highest daily dose of clozapine, we used linear regression models accounting for relevant demographic, pharmacological, and treatment covariates. In our main analysis, these included sex, age, and age^2^, all present in the CLOZUK and Norwegian records, and PRS metrics as proxies for body mass index, coffee intake, and smoking habits. As the data on the Norwegian cohort included explicit information on smoking habits, analyses of this cohort also explored the effects of including these data in the regression model, independently and in conjunction with the smoking behavior PRS. All regression models were built in the statistical software R version 4.1.0. The change in *R*^2^ owing to the inclusion of each covariate (also known as semipartial *R*^2^ or Δ*R*^2^) was estimated as an index of the proportion of variance explained by any individual factor in our model using the rockchalk package (31).

In further analyses, we also expanded our models by including other predictors that might affect the highest dose outcome, and which could potentially act as mediators of our observed effects. The clozapine plasma concentrations and the clozapine/norclozapine metabolic ratio observed at the point of highest dose were evaluated as well as the frequency of monitoring assessments (Supplemental Results).

To account for potential confounding from population stratification, we included the probabilities of pertaining to 4 of our possible 5 biogeographical groups in all CLOZUK regression models (Supplemental Methods). The first 10 principal components (PCs) were also used as regression model covariates, both in CLOZUK and in the Norwegian TDM cohort.

#### Secondary Analyses

We also undertook a series of analyses in the CLOZUK cohorts focusing on a broad but clinically relevant categorization of clozapine dose. We used multinomial and binary logistic regression models to estimate the effects of the schizophrenia PRS in the probability of taking the highest clozapine dose within 3 different dose groups: low (<300 mg/day), standard (300–600 mg/day), and high (>600 mg/day) (23). We fitted 3 separate pairwise regression models to assess differences between groups, using the same covariates in these models as in the primary analyses. In addition, to ensure compatibility between the CLOZUK2 and CLOZUK3 PRSs and PC analysis variables, we used the deduplicated PGC summary statistics as the PRS training set in this secondary analysis and generated the scores and PCs on strictly overlapping markers passing all quality control filters in the merged sample. As part of sensitivity analysis, we also collapsed individuals taking doses in the low and standard ranges into one category and compared them with those taking high doses (>600 mg/day) because prescribing a high clozapine daily dose generally requires more complex clinical considerations given the likelihood of ADRs than switches within lower thresholds. In this model, we calculated the area under the curve from receiver operating characteristic curves using the pROC package (32) in R. This is as a rough estimate of the added utility of our genetic predictor when combined with standard demographic variables used in clinical prediction modeling (33,34).

## Results

### Primary Analyses: Association of the Schizophrenia PRS and the Highest Clozapine Dose

**Figure 2:**
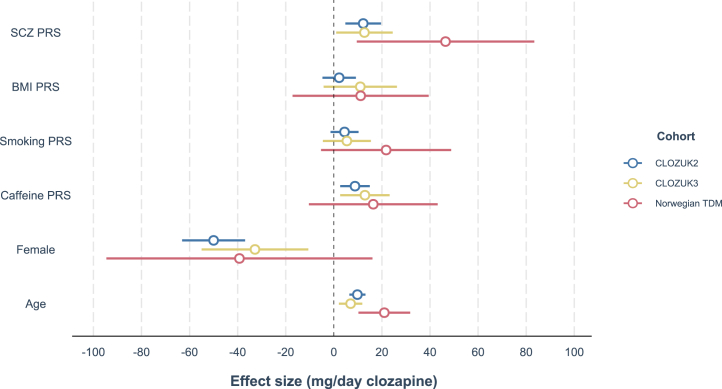
Effect size estimates for the main predictors of highest clozapine daily dose. Effects are represented as mean coefficient estimates (± 95% confidence intervals) for CLOZUK2 (blue), CLOZUK3 (yellow), and the Norwegian TDM sample (red). BMI, body mass index; PRS, polygenic risk score; SCZ, schizophrenia; TDM, therapeutic drug monitoring.

We observed a positive correlation between the schizophrenia PRS and the highest clozapine dose in our largest sample, CLOZUK2 (β = 12.217, 95% CI, 4.816–19.618, *p* = .001), where the variance explained by the schizophrenia PRS was Δ*R*^2^ ∼ 0.32%. Effect sizes expressed as the change in clozapine dose (mg/day) for 1-unit increase of the main predictors, accounting for other model covariates, can be seen in Figure 2. These results were essentially unchanged when accounting for possible mediators such as clozapine plasma concentrations, clozapine/norclozapine ratio, the frequency of clozapine monitoring in our dataset, and genetic variants known to affect CYP1A2 metabolism (Tables S2–S6). In addition, we explored whether the schizophrenia PRS was correlated with other features of clozapine metabolism, but no significant associations were found between the PRS and clozapine plasma concentrations or the clozapine/norclozapine ratio (Table S6).

Exploring our results further via sensitivity analyses, we saw little change in our CLOZUK2 schizophrenia PRS association by restricting the sample to individuals of European genetic ancestry (*n* = 2577, β = 11.46, 95% CI, 3.169–19.75, *p* = .007) and established that this result is specific to schizophrenia genetic liability by assessing a wider range of psychiatric, cognitive, and personality PRSs, none of which were significantly associated with clozapine doses (Figure S2).

To validate our findings, we replicated this analysis in 2 independent datasets. In CLOZUK3, we found results of similar magnitude and sign (β = 12.730, 95% CI, 0.996–24.464, *p* = .033, Δ*R*^2^ ∼ 0.48%) (Figure 2B), even after controlling for possible mediators (Table S2). In the Norwegian cohort, the results showed the same direction of effect with a larger magnitude and confidence interval (β = 46.451, 95% CI, 9.424–83.477, *p* = .014, Δ*R*^2^ ∼ 1.33%) (Figure 2), consistent with the smaller size of this dataset. In any case, all Norwegian effect sizes were still within the confidence interval range observed in the CLOZUK2 discovery analyses, even when controlling for possible mediators (Table S4). Moreover, these results were consistent when replacing the smoking behavior PRS with directly assessed smoking habits (β = 47.759, 95% CI, 10.817–84.700, *p* = .011, Δ*R*^2^ ∼ 1.41%) (Table S5).

### Secondary Analyses: Genetics-Informed Classification Model of Clozapine Doses

**Figure 3:**
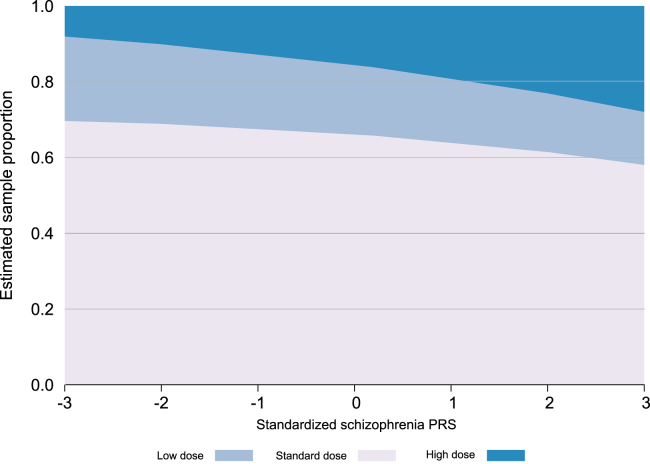
Probability estimates for each of the highest clozapine dose categories according to the schizophrenia PRS. The y-axis represents the probability of belonging to each of the 3 dose groups for individuals in the PRS spectrum. PRS, polygenic risk score.

We next explored to what extent the schizophrenia PRS could reflect broad clozapine prescription patterns in the complete CLOZUK cohort by using a multinomial regression model (Figure 3). For this, in stratified analyses by clozapine dose categories, we observed that the schizophrenia PRS was associated with the probability of taking high doses when compared with those taking either standard doses (odds ratio = 1.277, 95% CI, 1.066–1.530, *p* = .008) or low doses (odds ratio = 1.280, 95% CI, 1.029–1.593, *p* = .027).

**Figure 4:**
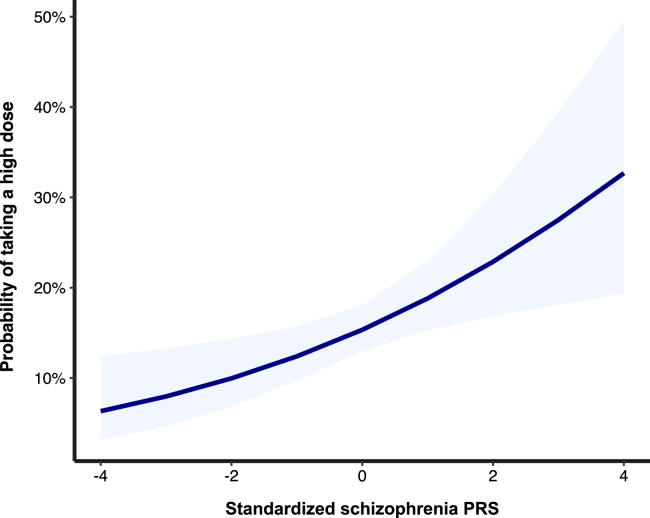
Probability of taking a high clozapine daily dose at different levels of schizophrenia PRS, represented using a logit function (banded area shows 95% confidence interval). PRS, polygenic risk score.

A second stratified analysis specifically examined differences in those taking clozapine doses over 600 mg/day against those below this threshold. In this analysis, we observed an association between the schizophrenia PRS and the probability of taking high doses (odds ratio = 1.279, 95% CI, 1.076–1.522, *p* = .005). These results are shown in Table 1 and as a logit probability curve in Figure 4. As an illustration of the detected effects, while the overall prevalence of individuals taking a high dose of clozapine was 15% in the entire CLOZUK sample, it surpassed 20% among those more than 2 standard deviations above the mean on the schizophrenia PRS, reaching 30% at the upper end of the PRS distribution.

**Table 1:** Effect Sizes of Each Predictor Included in the Model in Relation to the Probability of Taking a High Clozapine Dose

Predictor	Odds Ratio	2.5% CI	97.5% CI	*p*
PRS SCZ	1.279	1.076	1.522	.005
PRS BMI	1.025	0.824	1.275	.825
PRS Smoking	0.969	0.837	1.122	.677
PRS Coffee	1.152	0.988	1.344	.071
Age	1.105	1.006	1.215	.038
Age^2^	0.999	0.998	1.000	.041
Female	0.587	0.406	0.849	.005
PC1	1.272	0.803	2.014	.306
PC2	0.671	0.333	1.352	.264
PC3	0.856	0.532	1.376	.520
PC4	1.086	0.799	1.476	.600
PC5	1.177	0.955	1.452	.127
PC6	1.048	0.877	1.254	.605
PC7	1.273	0.985	1.644	.065
PC8	1.024	0.859	1.220	.794
PC9	0.712	0.527	0.963	.028
PC10	1.093	0.926	1.291	.292
SSA	1.001	0.985	1.018	.903
SAS	1.001	0.977	1.025	.952
EAS	1.014	0.951	1.080	.675
NEA	1.008	0.993	1.022	.295
Batch	1.075	0.740	1.561	.704

BMI, body mass index; EAS, East Asia; NEA, North Africa/Near East; PC, principal component; PRS, polygenic risk score; SAS, Southwest Asia; SCZ, schizophrenia; SSA, Sub-Saharan Africa.

Finally, we assessed the sensitivity and specificity of our prediction models for high clozapine doses by calculating the area under the curve from receiver operating characteristic statistics (presented in Figure S3). The area under the curve from the model including all covariates in the previous analysis (Figure 4 and Table 1) was 0.64, while for the demographics-only model (not including any genetically derived covariate), it was 0.58. We also showed that even when accounting for clozapine plasma concentrations, a known target of dose optimization for clozapine and a strong correlate of actual doses, the inclusion of genetic information marginally improved prediction accuracy.

## Discussion

This study examined whether the polygenic risk for schizophrenia would be associated with the daily dose of clozapine in 3 independent TRS cohorts, 2 with individuals from multiple ancestries. Our main result demonstrates an association between genetic liability for schizophrenia indexed by the PRS and the highest dose of clozapine available in treatment records. Furthermore, this effect is independent of known genetic factors associated with clozapine metabolism (Table S6). In the secondary analysis, individuals with a high genetic risk of the disorder had a 2-fold increased probability of taking high doses (>600 mg/day) compared with those on the lower end of the schizophrenia PRS spectrum (Figure 3). To our knowledge, this is the first study to report these associations. A recent investigation assessed the relationship between schizophrenia PRSs and clozapine doses but did not find any significant linear effects. The sample size (*n* = 44), though, was very limited compared with ours (22).

Identifying individuals who are more or less likely to respond to different pharmacological treatments has long been one of the hoped-for applications of PRSs in precision medicine (11,34). In this study, we leveraged the longitudinal aspect of clozapine monitoring and TDM and examined the highest clozapine daily dose recorded for each individual in these systems. We show that individuals in the high end of the schizophrenia PRS spectrum are more likely to be prescribed higher clozapine doses than the usual maintenance thresholds (300–600 mg/day). Taking this observation at face value implies that these individuals might have needed such high doses to obtain a therapeutic response from the outset of treatment, implying that genetic information could be used to personalize and plan clozapine prescriptions. However, the data available in our samples do not allow us to define the exact role or weight that genetic predictors should have for this potential application because it cannot be used to formally test the putative causal link between high clozapine doses and response to treatment. It is also uncertain whether individuals requiring high doses of clozapine reflect poorer responders (at low/standard doses) or those who might never respond to the drug, although a combination of both possibilities is likely (16). Indeed, Frank *et al.* (35) reported that individuals who were nonresponders to clozapine had the highest schizophrenia PRS in a TRS cohort. This result would also be consistent with our findings and raises the question as to the utility of clozapine for at least a subset of the individuals at the upper end of the schizophrenia PRS spectrum. Nevertheless, if more consistent and detailed evidence accumulates on the interplay between clozapine prescriptions, genetics, and treatment response, interventions might be devised to leverage this information with the goal of improving the overall tolerability and safety of the drug. This might also help address and prevent clozapine resistance, a severe and currently unpredictable outcome with no evidence-based treatment options (36).

Our study findings suggest that although we observed statistically significant associations, the variance explained by the schizophrenia PRS is small, and other genomic and nongenomic factors must contribute to a larger extent to the final phenotype. Indeed, we used several types of genetic predictors in our models (e.g., PCs, genetic ancestry, and PRSs for more than 1 trait), which combined to help explain a nontrivial amount of variance in the highest clozapine dose. This is in line with the notion that PRSs alone will likely have a relatively small impact in driving clinical practice even after their practical implementation becomes feasible (11). Knowing that a very complex network of factors affects antipsychotic response, we recommend caution in the interpretation of our findings’ potential clinical relevance, which needs to be further evaluated. Nevertheless, it has been shown in other areas such as cardiovascular disease that combining genetic information with nongenetic predictors and risk factors could be clinically meaningful and may help guide treatment choices (37). Once larger datasets become available, it would be beneficial to evaluate the use of PRSs as predictors for potentially stratified medicine approaches in a clinical trial. This could provide an opportunity to address whether individuals who carry a high schizophrenia PRS and adhere to clozapine therapy in the absence of adverse side effects should be offered alternative clozapine prescribing protocols when their response is suboptimal.

### Strengths and Limitations

The strengths of our study include its large sample size, as well as our taking advantage of some of the largest TRS cohorts in the world with genetic and longitudinal pharmacokinetic information. In addition, all the individuals with available data were used in our analyses regardless of ancestry, thus reflecting non-European populations that are traditionally underrepresented in genomics research. Another distinctive feature is the consistency and replication of the main finding across 3 different datasets. These included a Norwegian TDM cohort with reliable smoking data, which is a major pharmacokinetic determinant of clozapine and a potential confounder of no apparent relevance to the detected effects.

However, several limitations of this study need to be considered, and the results should be interpreted in light of these. First, our largest samples (CLOZUK2 and CLOZUK3) are based on electronic health records collected during mandatory clozapine monitoring, which do not include contextual information on clinical management, treatment response, or lifestyle. This affected our ability to determine factors known to influence clozapine metabolism in our study participants, including smoking status, weight, regular caffeine use, and the use of other medications. However, we attempted to mitigate these issues by using PRSs to derive genetically informed proxies of these as in a previous study (21), and these indeed contributed to explaining a part of the variance in our dataset and in the independent Norwegian TDM cohort (Tables S2–S5). Second, as in all retrospective analyses, unmeasured confounders might have had an influence on the effects detected, although all models were adjusted for known potential confounders in primary and sensitivity analyses. Third, we acknowledge that even though we presented evidence for an association of the schizophrenia PRS with clozapine dose in 3 independent samples, further research will require additional data on treatment response to evaluate mechanisms linking our observations to real-world prescribing practices.

As a final consideration, our main predictor is a schizophrenia PRS built from a mostly cross-sectional case-control analysis, which is not necessarily representative of the diversity of individuals and symptom profiles encompassed by real-world samples of those with schizophrenia or TRS. Moreover, although 2 of our 3 cohorts are multiancestry by design and were ascertained through a population-level clozapine monitoring system, they are all primarily composed of European individuals, and mainly European GWASs have been used in generating the PRS. For these reasons, it is difficult to evaluate whether potential downstream applications of our research would be translatable or broadly applicable to individuals from worldwide ethnic and genetic backgrounds.

In conclusion, we report that the schizophrenia PRS is associated with the highest clozapine dose on record in patients with TRS in 3 independent multiancestry cohorts, suggesting that genetic susceptibility to schizophrenia is associated with treatment choices in these samples. In the ongoing debate over the clinical utility of PRSs in precision psychiatry, our study adds to the growing body of evidence showing that genomic information can lead to novel answers to topics of interest for clinical care (11,38). More studies are needed to confirm our findings and to benchmark to what extent this or similar data could lead to future improvements in therapeutic decision making and in the overall clinical management of TRS.

## Supplementary Material

Key Resources Table
