# Supplementary material for: Genomic Stratification of Clozapine Prescription Patterns Using Schizophrenia Polygenic Scores
Source: Biol Psychiatry. 2023 Jan 15;93(2):149–56. doi: 10.1016/j.biopsych.2022.07.014 (PMC10804961; doi:10.1016/j.biopsych.2022.07.014)
Supplement: Supplementary Material [file mmc2.pdf]

SUPPLEMENT

Genomic Stratification of Clozapine Prescription Patterns Using Schizophrenia Polygenic Scores

Kappel *et al.*

Supplementary Note 2
Supplementary Methods 2
CLOZUK and Norwegian TDM genotyping and quality control 2
Genetic Ancestry assignment 2
Supplementary Results 3
Prediction modelling and AUC 3
Sensitivity analyses and potential confounders 3
Supplementary Tables 5
Supplementary Table 1 5
Supplementary Table 2 6
Supplementary Table 3 7
Supplementary Table 4 8
Supplementary Table 5 9
Supplementary Table 6 10
Supplementary Figures 11
Supplementary Figure 1 11
Supplementary Figure 2 12
Supplementary Figure 3 13
Supplementary References 14

Supplementary Note

Supplementary Methods

CLOZUK and Norwegian TDM genotyping and quality control

Detailed information regarding CLOZUK2 genotyping and quality control has been provided previously (1,2). For the new CLOZUK3 sample, genotype data was processed in the DRAGON-Data pipeline (3), using the *"GenotypeQCtoHRC"* module prior to imputation. Briefly, this pipeline includes basic genotypic quality control (QC) allowing for 5% of missing individual and marker data (4), checks for sample identifiability including the sex chromosomes (5), and assessment of genotyping errors by removal of markers outside Hardy-Weinberg equilibrium (mid p-value $<10^{-6}$) (6). Afterwards, imputation was performed on the Michigan Imputation Server with the Haplotype Reference Consortium (HRC) reference panel and default options (7).

For the merging of CLOZUK2+3 genomic data, we also followed the best practices implemented in *"GenotypeQCtoHRC"*, including the removal of all strand-ambiguous or non-overlapping SNPs and the recalculation of MAF thresholds in the whole sample.

For the Norwegian TDM cohort, DNA was extracted from whole blood and genotyped with Illumina® Human Omni Express-24 v.1.1 arrays at deCODE Genetics (Reykjavik, Iceland), following standard protocols from the array manufacturer. Standard pre-imputation quality control was performed using PLINK v1.9 (8). Chromosome-wide haplotypes were phased with Eagle2 (9,10) and missing variants were imputed with Minimac3 (7) using the first release of the HRC panel. After imputation, we removed variants with MAF<1% or HWE p-value $<10^{-6}$.

Genetic Ancestry assignment

Due to the diverse ethnic makeup of the CLOZUK study (1), all CLOZUK2 individuals previously underwent a genetic ancestry analysis using Ancestry Informative Markers (AIMs) in a Linear Discriminant Analysis (LDA) framework (11). We reproduced this model in CLOZUK3, deriving LDA-based probabilities of each individual belonging to five biogeographic regions - Europe, East Asia (EAS), Southwest Asia (SAS), North Africa (NEA) and Sub-Saharan Africa (SSA). The individual-level probabilities of pertaining to the biogeographical groups were used as covariates in all our CLOZUK regression models to account for potential confounding from population stratification, alongside 10 principal components generated with PCAiR (12). Note that since the five biogeographical probabilities necessarily sum to one, only four were included in our models (the European probability was omitted) to avoid collinearity with the regression intercept (13). **Supplementary Table 1** details the proportion of individuals classified as pertaining to each of those biogeographical groups.

Supplementary Results

Prediction modelling and AUC

In order to evaluate the added utility of using genetic predictors in our models, we calculated the area under the curve (AUC) from receiver operating characteristic (ROC) curves using the pROC package (14) in R . We compared this AUC with the analogous estimate from a model including only demographics (i.e., age, age^2, sex, and self-reported ethnicity), and another model including demographics and clozapine plasma concentrations to quantify the increase in predictive ability provided by genetic information alone (15,16). All the AUC values were calculated exclusively on the CLOZUK2 individuals to take advantage of the self-reported ethnicity information only available for this sample. This has been considered an important predictor for clozapine dose optimization (17) and is not always directly comparable to genetic ancestry estimates. These analyses are shown in **Supplementary Figure 3**.

Sensitivity analyses and potential confounders

We also evaluated the effects of including additional predictors that could potentially act as mediators of the association between schizophrenia PRS and highest clozapine dose. The first additional factor considered was the clozapine plasma concentration (or "levels"), observed at the point of highest dose (**Supplementary Tables 2-5**). A moderating effect of these concentrations would be consistent with schizophrenia risk variants reflecting features of drug metabolism, a hypothesis that has been explored in previous research on TRS (18). In the same sense, we also assessed the clozapine/norclozapine metabolic ratio (**Supplementary Table 6**), thought to reflect clozapine metabolism primarily through CYP1A2 activity and treatment adherence (19,20).

Another predictor included in these analyses was the number of times each individual's clozapine levels were assessed in the complete dataset. This could be a proxy for potential confounders as, to monitor adherence and anticipate level-dependent ADRs, pharmacokinetic monitoring or TDM are likely to be requested more frequently as individuals' clozapine doses escalate, thus increasing the likelihood of those on high doses being present in our dataset. Interestingly, although there was not an obvious linear effect between the monitoring frequency and the schizophrenia PRS either (**Supplementary Table 2**), we did observe larger clozapine daily doses in individuals who underwent monitoring more frequently (**Supplementary Figure 1**), aligned with our initial expectations.

We also confirmed that our main detected effects were still consistent and significant when restricting our dataset to observations at clozapine plasma concentration thresholds investigated in previous research on therapeutic response to the drug (21), either between 350-600 ng/L (n= 1089; β= 14.952, 95% CI: [3.167-26.737], P= 0.0131) or above (n= 1283; β= 17.258, 95% CI [5.823-28.692], P= 0.0031).

Finally, we evaluated if genetic variants known to affect CYP1A2 metabolism, the main enzyme involved in clozapine first pass-metabolism could partially explain the effects we observed.

Therefore, we assessed the counts of the *CYP1A2**1F (rs762551_A) allele (22,23), used it to define Normal or Ultra-rapid CYP1A2 metabolizers, and included this information as a covariate in the regression models of our largest cohort (merged CLOZUK2 and CLOZUK3, as in "Secondary Analyses" in the main text). The inclusion of this covariate did not affect the results we first observed (**Supplementary Table 6**). Similarly, we also show that the inclusion of the rs2472297_T SNP, located in the intergenic *CYP1A1* and *CYP1A2* region and previously shown to be associated with clozapine and olanzapine plasma concentrations (2,24), does not change the reported association of schizophrenia PRS and clozapine dose (**Supplementary Table 6**).

Moreover, we considered that the effects of schizophrenia PRS could be partially explained by the effects of genetic variants acting on clozapine metabolism being included in the PRS. Given this, we derived new schizophrenia PRS scores after the removal of genes that could impact clozapine metabolism. **Supplementary Table 6** shows that schizophrenia PRSs that do not contain any of the 18 genes in the PharmGKB clozapine pathway (25) - 558814 SNPs included), or the 59 CPIC Very Important Pharmacogenes -Tier 1 and Tier 2 (https://www.pharmgkb.org/vips - 558027 SNPs included), are associated with the highest clozapine dose, and the magnitude of effect is fundamentally unchanged from the genome-wide PRS (559297 SNPs).

Supplementary Tables

Supplementary Table 1: Descriptive statistics for demographic and clinical variables for each sample used.

		CLOZUK2		CLOZUK3		NORWEGIAN TDM	
		Mean	SD	Mean	SD	Mean	SD
Age		42.98	11.10	42.97	11.08	40.62	14.08
Highest clozapine dose (mg/day)		428.89	167.19	429.47	167.22	476.78	280.93
Clozapine concentration (ng/mL)		597.06	369.41	597.29	369.96	474.80	336.41
Frequency of assessments		3.56	3.53	3.34	2.66	19.10	20.76
		N	%	N	%	N	%
Sex							
	Males	2302	73.48	655	72.06	259	62.11
	Females	831	26.52	254	27.94	158	37.89
Ancestry							
	Admixed/Unknown	84	2.68	38	4.18	-	-
	East Asian	31	0.99	5	0.55	-	-
	European	2577	82.25	761	83.72	417	100
	North African	92	2.94	26	2.86	-	-
	Southwest Asian	173	5.52	42	4.62	-	-
	Sub-Saharan African	176	5.62	37	4.07	-	-

Supplementary Table 2: Linear regression model for highest clozapine daily dose adjusting for potential confounders in CLOZUK2

	Beta	S.E.	P	ΔR^2
PRS SCZ	11.348	3.597	0.002	0.0027
age	8.973	1.626	3.71E-08	0.0084
age^2	-0.103	0.018	1.19E-08	0.0090
Female	-57.016	6.383	7E-19	0.0219
PRS BMI	3.672	3.374	0.277	0.0003
PRS smoking	5.032	2.829	0.075	0.0009
PRS coffee	11.181	2.991	0.000	0.0038
PC1	1.746	11.780	0.882	<0.0001
PC2	22.807	11.342	0.044	0.0011
PC3	-0.823	7.528	0.913	<0.0001
PC4	6.787	4.892	0.166	0.0005
PC5	0.304	2.919	0.917	<0.0001
PC6	0.363	2.984	0.903	<0.0001
PC7	6.192	2.161	0.004	0.0023
PC8	-1.295	1.695	0.445	0.0002
PC9	-1.968	3.233	0.543	0.0001
PC10	3.678	2.990	0.219	0.0004
EAS	1.331	1.046	0.204	0.0004
SAS	-0.192	0.374	0.607	0.0001
SSA	-0.135	0.458	0.768	<0.0001
NEA	0.106	0.311	0.733	<0.0001
clozapine levels	0.104	0.008	6.67E-41	0.0508
assessments	7.436	0.802	3.35E-20	0.0236

Supplementary Table 3: Linear regression model for highest clozapine daily dose adjusting for potential confounders in CLOZUK3

	Beta	S.E.	P	ΔR^2
PRS SCZ	10.972	5.785	0.058	0.0035
age	5.899	2.400	0.014	0.0059
age^2	-0.073	0.028	0.009	0.0068
Female	-30.315	10.989	0.006	0.0075
PRS BMI	10.610	7.507	0.158	0.0020
PRS smoking	5.222	4.931	0.290	0.0011
PRS coffee	14.439	5.073	0.005	0.0080
PC1	-13.488	13.991	0.335	0.0009
PC2	-9.828	17.536	0.575	0.0003
PC3	15.708	8.700	0.071	0.0032
PC4	-5.526	15.133	0.715	0.0001
PC5	-0.194	6.854	0.977	<0.0001
PC6	14.061	5.732	0.014	0.0059
PC7	-3.537	4.899	0.471	0.0005
PC8	-10.104	5.078	0.047	0.0039
PC9	-3.342	4.893	0.495	0.0005
PC10	-5.942	4.646	0.201	0.0016
EAS	0.766	2.132	0.720	0.0001
SAS	-0.110	0.870	0.899	<0.0001
SSA	0.383	0.511	0.454	0.0006
NEA	0.591	0.616	0.337	0.0009
clozapine levels	0.044	0.016	0.006	0.0074
assessments	6.609	0.899	4.37E-13	0.0531

Supplementary Table 4: Linear regression model for highest clozapine daily dose adjusting for potential confounders in the Norwegian TDM cohort

	Beta	S.E.	P	ΔR^2
PRS SCZ	37.999	17.171	0.028	0.0088
age	9.798	5.124	0.057	0.0066
age^2	-0.132	0.055	0.018	0.0102
Female	-52.051	25.674	0.043	0.0074
PRS BMI	9.313	13.108	0.478	0.0009
PRS smoking	35.865	12.648	0.005	0.0145
PRS coffee	5.672	12.465	0.649	0.0004
PC1	64.681	22.982	0.005	0.0143
PC2	-41.567	22.599	0.067	0.0061
PC3	16.303	19.583	0.406	0.0013
PC4	-8.795	16.430	0.593	0.0005
PC5	13.165	13.093	0.315	0.0018
PC6	-0.305	13.503	0.982	<0.0001
PC7	13.232	12.383	0.286	0.0021
PC8	-24.431	14.584	0.095	0.0051
PC9	-5.857	14.032	0.677	0.0003
PC10	-0.756	13.805	0.956	<0.0001
batch	17.644	26.842	0.511	0.0008
clozapine levels	0.254	0.040	3.78E-10	0.0745
assessments	3.000	0.644	4.32E-06	0.0392

Supplementary Table 5: Linear regression model for highest clozapine daily dose adjusting for potential confounders and for smoking status in the Norwegian TDM cohort

	Beta	S.E.	P	ΔR^2
PRS SCZ	39.930	16.928	0.019	0.0098
age	4.839	5.192	0.352	0.0015
age^2	-0.078	0.056	0.166	0.0034
Female	-39.912	25.538	0.119	0.0043
smoking status	115.572	26.381	1.52E-05	0.0337
PRS BMI	5.092	12.993	0.695	0.0003
PRS coffee	9.584	12.128	0.430	0.0011
PC1	58.981	22.583	0.009	0.0120
PC2	-38.719	22.310	0.083	0.0053
PC3	18.368	19.327	0.343	0.0016
PC4	-9.757	16.209	0.548	0.0006
PC5	13.886	12.909	0.283	0.0020
PC6	-0.910	13.318	0.946	<0.0001
PC7	11.601	12.203	0.342	0.0016
PC8	-23.649	14.388	0.101	0.0047
PC9	0.400	13.717	0.977	<0.0001
PC10	0.929	13.616	0.946	<0.0001
batch	8.013	26.624	0.764	0.0002
clozapine levels	0.276	0.040	1.23E-11	0.0857
assessments	3.128	0.636	1.27E-06	0.0425

Supplementary Table 6: Additional results detailing the effects of the schizophrenia PRS on highest daily dose in the merged CLOZUK sample (2+3)

	**PRS SCZ**		
PRS calculated on different genomic backgrounds	Beta	SE	P-value
Primary analyses (genome-wide)	11.882	2.817	3.00E-05
No clozapine pathway genes	10.814	2.882	1.80E-04
No CPIC VIP genes	10.311	2.866	3.30E-04
New covariates included in the original model	Beta	SE	P-value
Clozapine/norclozapine ratio	11.378	2.782	4.00E-05
*CYP1A2**1F (Ultra-rapid metabolizers)	12.535	2.828	1.00E-05
CYP1A2 (rs2472297_T)	12.469	2.818	1.00E-05
Association with other phenotypes	Beta	SE	P-value
Clozapine plasma concentration	1.621	6.382	0.7995
Clozapine/norclozapine ratio	-0.013	0.012	0.2604

Regression coefficients (mg/day clozapine) are presented only for the schizophrenia PRS; however, all analyses were adjusted for the same covariates included in Supplementary Tables (2-3).

PRS SCZ = schizophrenia polygenic risk score (26)
Clozapine pathway: https://www.pharmgkb.org/pathway/PA166163661/components
CPIC VIP genes = Clinical Pharmacogenetics Implementation Consortium's Very Important Pharmacogenes (Tier 1 and 2): https://www.pharmgkb.org/vips
*CYP1A2**1F: https://www.pharmvar.org/gene/CYP1A2
CYP1A2 (rs2472297_T): (2,24)

Supplementary Figures

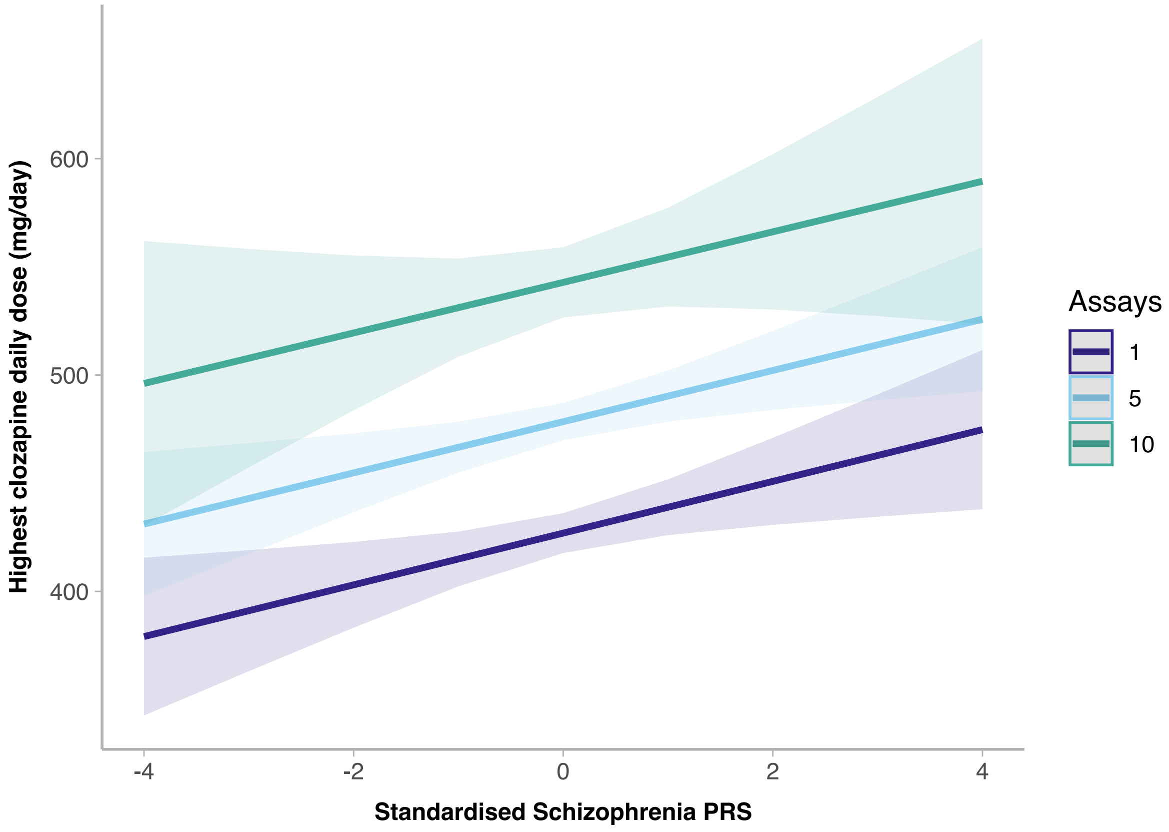

Supplementary Figure 1 - Effect of clozapine monitoring frequency on the relationship between schizophrenia PRS and highest clozapine daily dose in CLOZUK2. PRS = polygenic risk score

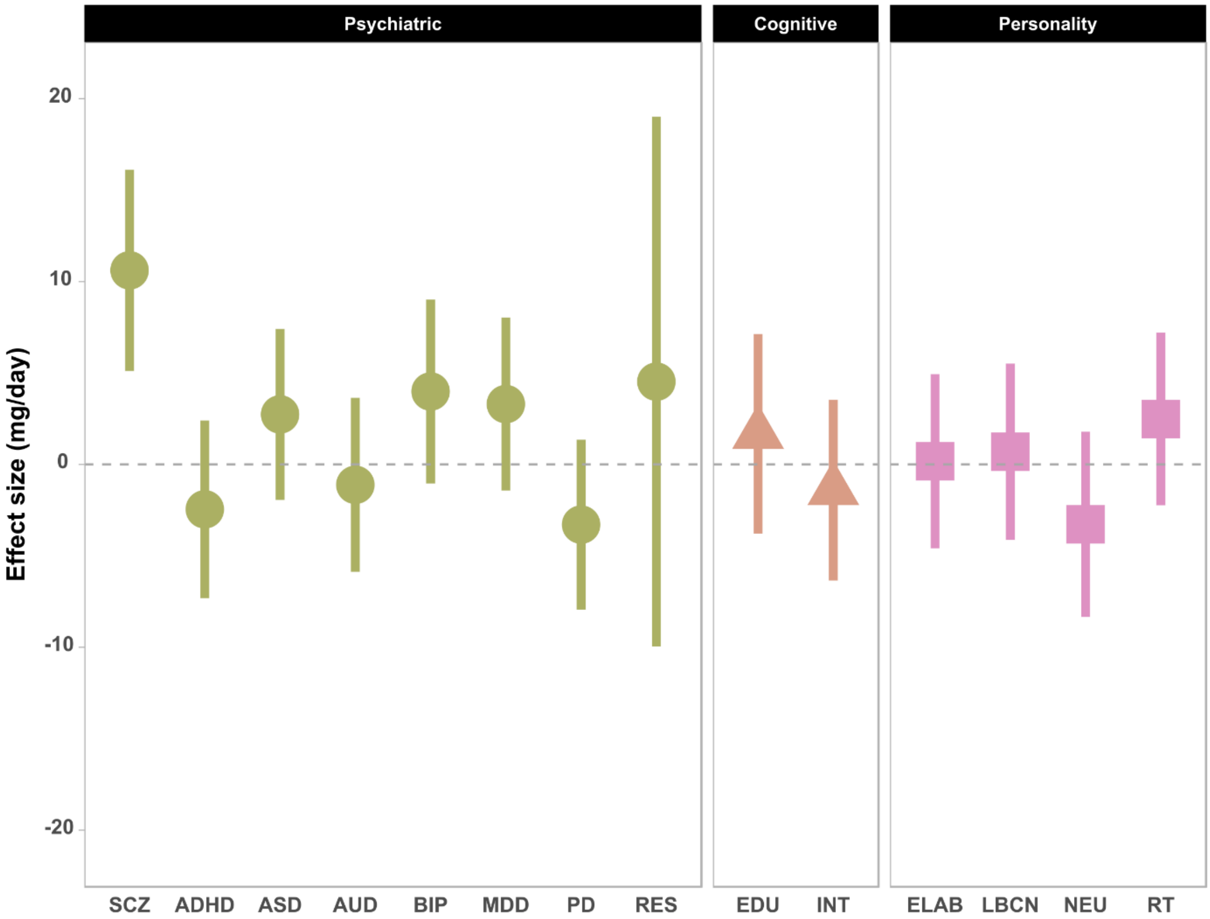

Supplementary Figure 2 – Association of psychiatric, cognitive and personality PRS with highest clozapine doses. Underlying regressions use the same full model covariates as Supplementary Tables 2-3 and were computed on the merged CLOZUK2+3 sample for increased statistical power. All PRS were computed with the PRScs software (see methods).

SCZ: Schizophrenia (n= 130300) (26)
ADHD: Attention deficit hyperactivity disorder (n= 55374) (27)
ASD: Autism spectrum disorder (n= 46350) (28)
AUD: Alcohol use disorder (n= 121604) (29)
BIP: Bipolar disorder (n= 413466) (30)
MDD: Major depressive disorder (n= 807553) (31)
PD: Panic disorder (n= 10240) (32)
RES: Resilience to schizophrenia (n= 22405) (33)
EDU: Educational attainment (n= 765283) (34)
INT: Intelligence (n= 269867) (35)
ELAB: Emotional lability (n= 5133) (36)
LBCN: Lack of behavioral control (n= 5133) (36)
NEU: Neuroticism (n= 582989) (37)
RT: Risk-taking behavior (n= 939908) (38)

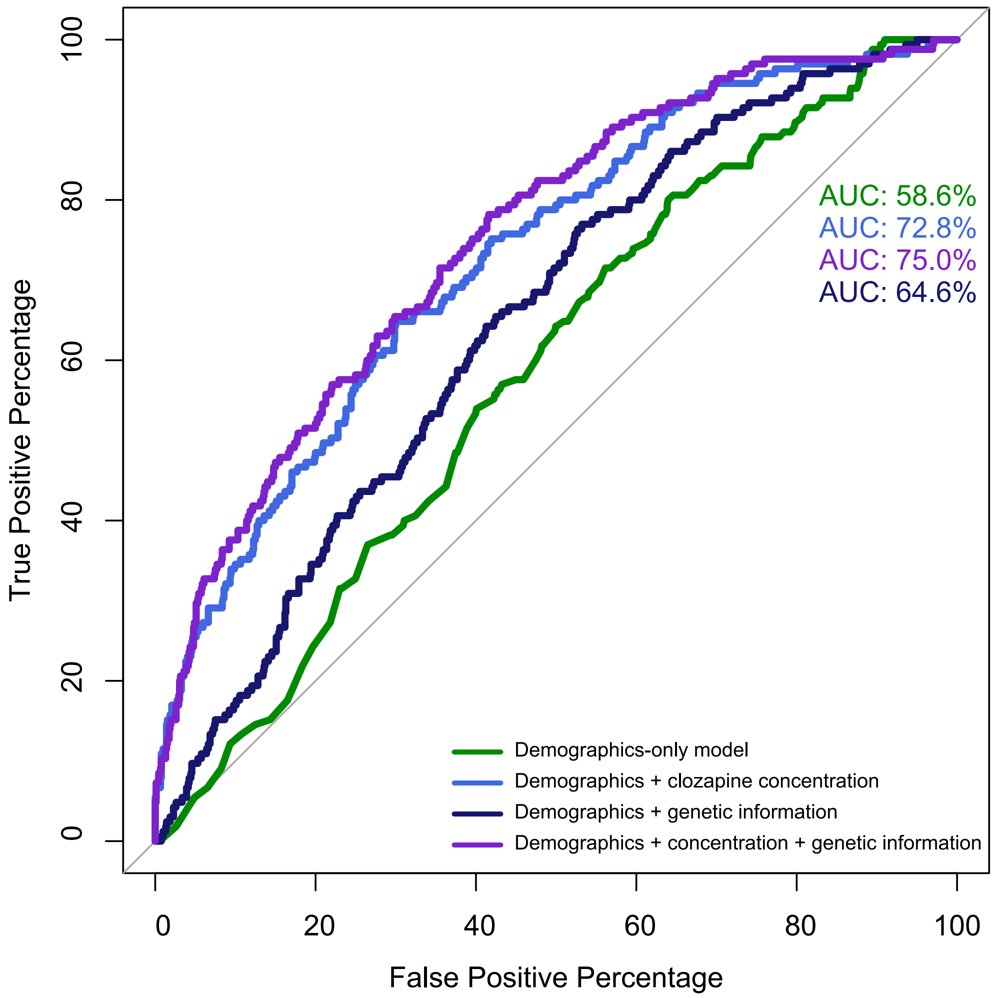

Supplementary Figure 3 – ROC curves for prediction accuracy of probability of taking a high dose from models with different levels of information in CLOZUK2.

Demographics-only model included = sex, age, age^2 and self-reported ethnicity

Demographics + clozapine included = sex, age, age^2, self-reported ethnicity and clozapine plasma concentration

Demographics + genetics included = sex, age, age^2, BMI PRS, Smoking PRS, Caffeine PRS, 10 genetic principal components and the probabilities of belonging to four biogeographic regions (EAS, SAS, SSA, NEA)

Demographics+ clozapine concentration + genetic information included a full prediction model: sex, age, age^2, BMI PRS, Smoking PRS, Caffeine PRS, 10 genetic principal components, the probabilities of belonging to each biogeographic region (EAS, SAS, SSA, NEA) and clozapine concentration levels

Supplementary References

1. Pardiñas AF, Holmans P, Pocklington AJ, Escott-Price V, Ripke S, Carrera N, *et al.* (2018): Common schizophrenia alleles are enriched in mutation-intolerant genes and in regions under strong background selection. *Nat Genet* 50: 381–389.

2. Pardiñas AF, Nalmpanti M, Pocklington AJ, Legge SE, Medway C, King A, *et al.* (2019): Pharmacogenomic variants and drug interactions identified through the genetic analysis of clozapine metabolism. *Am J Psychiatry* 176: 477–486.

3. Hubbard L, Lynham AJ, Knott S, Underwood JFG, Anney R, Bisson JI, *et al.* (2022): DRAGON-Data: A platform and protocol for integrating genomic and phenotypic data across large psychiatric cohorts. *medRxiv* 2022.01.18.22269463.

4. Anderson CA, Pettersson FH, Clarke GM, Cardon LR, Morris AP, Zondervan KT (2010): Data quality control in genetic case-control association studies. *Nat Protoc* 5: 1564–1573.

5. Igo RP, Cooke Bailey JN, Romm J, Haines JL, Wiggs JL (2016): Quality Control for the Illumina HumanExome BeadChip. *Curr Protoc Hum Genet* 90. https://doi.org/10.1002/cphg.15

6. Graffelman J, Jain D, Weir B (2017): A genome-wide study of Hardy–Weinberg equilibrium with next generation sequence data. *Hum Genet* 136: 727–741.

7. Das S, Forer L, Schönherr S, Sidore C, Locke AE, Kwong A, *et al.* (2016): Next-generation genotype imputation service and methods. *Nat Genet* 48: 1284–1287.

8. Chang CC, Chow CC, Tellier LCAM, Vattikuti S, Purcell SM, Lee JJ (2015): Second-generation PLINK: Rising to the challenge of larger and richer datasets. *Gigascience* 4. https://doi.org/10.1186/s13742-015-0047-8

9. Loh P-R, Danecek P, Palamara PF, Fuchsberger C, A Reshef Y, K Finucane H, *et al.* (2016): Reference-based phasing using the Haplotype Reference Consortium panel. *Nat Genet* 48: 1443–1448.

10. Loh P-R, Palamara PF, Price AL (2016): Fast and accurate long-range phasing in a UK Biobank cohort. *Nat Genet* 48: 811–6.

11. Legge SE, Pardiñas AF, Helthuis M, Jansen JA, Jollie K, Knapper S, *et al.* (2019): A genome-wide association study in individuals of African ancestry reveals the importance of the Duffy-null genotype in the assessment of clozapine-related neutropenia. *Mol Psychiatry* 24: 328–337.

12. Conomos MP, Miller MB, Thornton TA (2015): Robust Inference of Population Structure for Ancestry Prediction and Correction of Stratification in the Presence of Relatedness. *Genet Epidemiol* 39: 276–293.

13. Gelman A, Hill J, Vehtari A (2020): *Regression and Other Stories*. Cambridge University Press. https://doi.org/10.1017/9781139161879

14. Robin X, Turck N, Hainard A, Tiberti N, Lisacek F, Sanchez J-C, Müller M (2011): pROC: an open-source package for R and S+ to analyze and compare ROC curves. *BMC Bioinformatics* 12: 77.

15. Hanley JA, McNeil BJ (1982): The meaning and use of the area under a receiver operating characteristic (ROC) curve. *Radiology* 143: 29–36.

16. Lewis CM, Vassos E (2020, May 18): Polygenic risk scores: From research tools to clinical instruments. *Genome Medicine*, vol. 12. BioMed Central Ltd., p 44.

17. de Leon J, Schoretsanitis G, Smith RL, Molden E, Solismaa A, Seppälä N, *et al.* (2021): An International Adult Guideline for Making Clozapine Titration Safer by Using Six Ancestry-Based Personalized Dosing Titrations, CRP, and Clozapine Levels. *Pharmacopsychiatry*. https://doi.org/10.1055/a-1625-6388

18. Ruderfer DM, Charney AW, Readhead B, Kidd BA, Kähler AK, Kenny PJ, *et al.* (2016): Polygenic overlap between schizophrenia risk and antipsychotic response: a genomic medicine approach. *The Lancet Psychiatry* 3: 350–357.

19. Costa-Dookhan KA, Agarwal SM, Chintoh A, Tran VN, Stogios N, Ebdrup BH, *et al.* (2020): The clozapine to norclozapine ratio: a narrative review of the clinical utility to minimize metabolic risk and enhance clozapine efficacy. *Expert Opin Drug Saf* 19: 43–57.

20. Ellison JC, Dufresne RL (2015): A review of the clinical utility of serum clozapine and norclozapine levels. *Ment Heal Clin* 5: 68–73.

21. Siskind D, Sharma M, Pawar M, Pearson E, Wagner E, Warren N, Kisely S (2021): Clozapine levels as a predictor for therapeutic response: A systematic review and meta-analysis. *Acta Psychiatr Scand* 144: 422–432.

22. Gaedigk A, Casey ST, Whirl-Carrillo M, Miller NA, Klein TE (2021): Pharmacogene Variation Consortium: A Global Resource and Repository for Pharmacogene Variation. *Clin Pharmacol Ther* 110: 542–545.

23. Gaedigk A, Ingelman-Sundberg M, Miller NA, Leeder JS, Whirl-Carrillo M, Klein TE (2018): The Pharmacogene Variation (PharmVar) Consortium: Incorporation of the Human Cytochrome P450 (CYP) Allele Nomenclature Database. *Clin Pharmacol Ther* 103: 399–401.

24. Söderberg MM, Haslemo T, Molden E, Dahl M-L (2013): Influence of CYP1A1/CYP1A2 and AHR polymorphisms on systemic olanzapine exposure. *Pharmacogenet Genomics* 23: 279–85.

25. Thorn CF, Müller DJ, Altman RB, Klein TE (2018): PharmGKB summary. *Pharmacogenet Genomics* 28: 214–222.

26. Trubetskoy V, Pardiñas AF, Qi T, Panagiotaropoulou G, Awasthi S, Bigdeli TB, *et al.* (2022): Mapping genomic loci implicates genes and synaptic biology in schizophrenia. *Nature* 604: 502–508.

27. Demontis D, Walters RK, Martin J, Mattheisen M, Als TD, Agerbo E, *et al.* (2019): Discovery of the first genome-wide significant risk loci for attention deficit/hyperactivity disorder. *Nat Genet* 51: 63–75.

28. Grove J, Ripke S, Als TD, Mattheisen M, Walters RK, Won H, *et al.* (2019): Identification of common genetic risk variants for autism spectrum disorder. *Nat Genet* 51: 431–444.

29. Sanchez-Roige S, Palmer AA, Fontanillas P, Elson SL, Adams MJ, Howard DM, *et al.* (2019): Genome-Wide Association Study Meta-Analysis of the Alcohol Use Disorders Identification Test (AUDIT) in Two Population-Based Cohorts. *Am J Psychiatry* 176: 107–118.

30. Mullins N, Forstner AJ, O'Connell KS, Coombes B, Coleman JRI, Qiao Z, *et al.* (2021): Genome-wide association study of more than 40,000 bipolar disorder cases provides new insights into the underlying biology. *Nat Genet* 53: 817–829.

31. Howard DM, Adams MJ, Clarke T-K, Hafferty JD, Gibson J, Shirali M, *et al.* (2019): Genome-wide meta-analysis of depression identifies 102 independent variants and highlights the importance of the prefrontal brain regions. *Nat Neurosci* 22: 343–352.

32. Forstner AJ, Awasthi S, Wolf C, Maron E, Erhardt A, Czamara D, *et al.* (2021): Genome-wide association study of panic disorder reveals genetic overlap with neuroticism and depression. *Mol Psychiatry* 26: 4179–4190.

33. Hess JL, Tylee DS, Mattheisen M, Børglum AD, Als TD, Grove J, *et al.* (2021): A polygenic resilience score moderates the genetic risk for schizophrenia. *Mol Psychiatry* 26: 800–815.

34. Okbay A, Wu Y, Wang N, Jayashankar H, Bennett M, Nehzati SM, *et al.* (2022): Polygenic prediction of educational attainment within and between families from genome-wide association analyses in 3 million individuals. *Nat Genet* 54: 437–449.

35. Savage JE, Jansen PR, Stringer S, Watanabe K, Bryois J, de Leeuw CA, *et al.* (2018): Genome-wide association meta-analysis in 269,867 individuals identifies new genetic and functional links to intelligence. *Nat Genet* 50: 912–919.

36. Heilbronner U, Papiol S, Budde M, Andlauer TFM, Strohmaier J, Streit F, *et al.* (2021): "The Heidelberg Five" personality dimensions: Genome-wide associations, polygenic risk for neuroticism, and psychopathology 20 years after assessment. *Am J Med Genet Part B Neuropsychiatr Genet* 186: 77–89.

37. Baselmans BML, van de Weijer MP, Abdellaoui A, Vink JM, Hottenga JJ, Willemsen G, *et al.* (2019): A Genetic Investigation of the Well-Being Spectrum. *Behav Genet* 49: 286–297.

38. Karlsson Linnér R, Biroli P, Kong E, Meddens SFW, Wedow R, Fontana MA, *et al.* (2019): Genome-wide association analyses of risk tolerance and risky behaviors in over 1 million individuals identify hundreds of loci and shared genetic influences. *Nat Genet* 51: 245–257.
